# Supplementary material for: Safety of bedside surgical tracheostomy during COVID-19 pandemic: A retrospective observational study
Source: PLoS One. 2020 Sep 30;15(9):e0240014. doi: 10.1371/journal.pone.0240014 (PMC7526872; doi:10.1371/journal.pone.0240014)
Supplement: Attachment — Submitted filename: REVISION COVID TRACHEOSTOMIES PARMA.docx [file pone.0240014.s001.docx]

**REVIEW OF RESERCH ARTICLE by Dr Cristina Martin-Villares. PLOS ONE**

“SAFETY OF BESIDE SURGICAL TRACHEOSTOMY DURIN COVID-19 PANDEMIC: a retrospective observational study”.

Edoardo Picetti Azienda. Ospedaliero-Universitaria di Parma Parma, ITALY

This is a very interesting work of a long series from a single centre in one of the hot-spots of the pandemic in Europe: the North of Italy. In particular it focusses in evaluating the safety of bedside 67 surgical tracheostomy in COVID-19 patients admitted to ICU. The results of no contagion after 65 tracheostomies without negative pressure room, a very good result: congratulations.

In my opinion the manuscript can be published after considering some comments

PERSONAL COMMENTS

Q1

In line 191, the authors reported that the median time from ICU admission to tracheostomy was 6.1 (± 2.1) days. With such an early strategy, more than 50% of critical intubation patients underwent tracheostomy. This is a very controversial topic, the timing of tracheostomy in intubated patients, and trials are impossible during COVID-19 pandemic. But with this aggressive strategy some patients could loose the opportunity of weaning without tracheostomy?

Q2

In line 191-192, investigators reported that at the last day of follow-up, 39

(59.1%) patients were discharged alive from ICU, while 18 (27.3%) were still in the ICU.

This successfully results needs more follow up with 27,3% of patients still under mechanical ventilation with tracheostomy.

Q3

In line 217, I can read that 71.2 % of tracheostomies were performed by intensivists. In Spain, open tracheostomies are performed by otolaryngologists ( or sometimes thoracic and maxillofacial surgeons ) and intensivists only perform percutaneous tracheostomies.

Q4

In lines 268-269, “To our knowledge, this 269 represents the largest study on this topic (bedside surgical tracheostomy) in COVID-19 patients” in A SINGLE INSTITUTION. A recent paper published in European Archives of Oto-Rhino-Laryngology (Outcome of 1890 tracheostomies for critical COVID‑19 patients: a national cohort study in Spain (https://doi.org/10.1007/s00405-020-06220-3) describes a larger number of tracheostomies in various institutions of our group)

Q5

We are agree in the low rate of complications in COVID-19 tracheostomies in our experience

Q6

In line 223-24, the investigators report that at ICU discharge, all patients (39/39) still had tracheostomy in place. I understand that there were no patients decannulated from 65 tracheostomies. In our experience, weaning is complex, but decannulation after weaning is almost the rule.

Q7

In line 283-285, the reasons why the authors decided to perform surgical tracheostomies, instead of percutaneous tracheostomies are controversial.

Q9

In Bibliography, I recommended to review another Italian series in discussion as:

Picin	University of Bologna	24 tracheostomies

DÁsciano	Santa Croce Pesaro	22 tracheostomies

Mattioli	University of Modena	28 tracheostomies
